# Views on oral health determinants as described by persons with continuous positive airway pressure-treated obstructive sleep apnoea: a qualitative study

**DOI:** 10.1186/s12903-023-03108-6

**Published:** 2023-06-20

**Authors:** Hanna Ahonen, Margit Neher, Eleonor I. Fransson, Anders Broström, Ulrika Lindmark

**Affiliations:** 1 Centre for Oral Health, School of Health and Welfare, Jönköping University, Jönköping, Sweden; 2 School of Health and Welfare, Jönköping University, Jönköping, Sweden; 3 Department of Clinical Neurophysiology, University Hospital Linköping, Linköping, Sweden; 4 Department of Health Sciences, Karlstad University, Karlstad, Sweden

**Keywords:** Determinants, Oral health, Obstructive sleep apnoea, CPAP-treatment

## Abstract

**Background:**

Oral diseases have been associated with cardiovascular diseases, and persons with continuous positive airway pressure [CPAP]-treated obstructive sleep apnoea [OSA] have an increased risk for negative consequences for both oral and general health. CPAP treatment is often life-long and adherence to treatment is essential. Xerostomia is a common side-effect which can lead to treatment abandonment. Oral health is a changeable part of our general health and well-being and exploring the views of oral health determinants from persons with experience of CPAP-treatment is important to prevent adverse oral health outcomes. The purpose of this study was to explore what persons with experience of CPAP-treated OSA view as determinants for their oral health.

**Methods:**

Eighteen persons with long-term experience of CPAP-treated OSA were purposively selected. Data were collected by semi-structured individual interviews. A code book based on the World Dental Federation’s [FDI] theoretical framework for oral health was developed and used to analyse the data using directed content analysis. The domains in the framework’s component driving determinants were used as pre-determined categories. Using the description of driving determinants as a guide, meaning units were extracted from the interview transcripts through an inductive approach. Then, by employing a deductive approach the code book was used to categorise the meaning units into the pre-determined categories.

**Findings:**

The views on oral health determinants described by the informants were compatible with the five domains in the component driving determinants in the FDI’s theoretical framework. Ageing, heredity, and salivation (biological and genetic factors), influences from family and the wider society (social environment), location and re-localisation (physical environment), oral hygiene habits, motivation, willingness to change, professional support (health behaviours), and availability, control, finances, and trust (access to care) were viewed as important oral health determinants by the informants.

**Conclusion:**

The study points to a variety of individual oral health-related experiences that oral healthcare professionals could consider when designing interventions to reduce xerostomia and prevent adverse oral health outcomes for persons undergoing long-term CPAP-treatment.

## Introduction

Recently, the World Dental Federation [FDI] proposed a new definition and theoretical framework of oral health, intending to move dentistry from the traditional focus on treatments towards oral health promotion and support [1]. The FDI’s definition and theoretical framework describe oral health as multifaceted and changeable, including oral diseases/conditions, pain and discomfort, as well as aspects of physical and psychosocial functions and abilities essential to our everyday life [2]. Oral health and general health and well-being are intertwined with determinants in daily life, but also with biomedical associations, for example there are connections among periodontal diseases and systemic or metabolic diseases/disorders such as cardiovascular diseases and obesity [3–5]. Thus, determinants for oral and general health could be targeted simultaneously as both are affected by the circumstances in which we live, work, and become older [2, 6, 7]. However, even if obstructive sleep apnoea [OSA] and periodontal diseases previously have been associated through systemic inflammation as well common risk factors from everyday life (e.g. ageing and tobacco use), the association between OSA and oral diseases has remained unclear. Few studies regarding dental caries have been performed [8], and there is a need for more research on OSA and periodontal disease [9, 10], but also the patient perspective on if and how OSA and CPAP-treatment might affect the oral health.

Characterised by repeated episodes of physical obstructions in the upper airway, OSA is defined as a sleep-related breathing disorder. Risk factors for OSA include obesity/overweight, male sex, postmenopausal state in women, and older age [11], and it is estimated to affect approximately 900 million persons globally [12]. The obstruction often causes heavy snoring and impedes the airflow completely (apnoea) or partially (hypopnoea), causing disturbances in the breathing process and desaturation, and is associated with cardiovascular diseases such as hypertension [11]. To describe the severity of OSA, the metric tool the Aponea Hypopnoea Index [AHI] is commonly used where AHI 5–14.9 indicates mild, 15–29.9 moderate, and ≥ 30 severe OSA. However, it is also important to consider comorbidities and the effect on everyday life when deciding treatment [11]. Continuous Positive Airway Pressure [CPAP] has been described as the primary choice of treatment for symptomatic OSA and, if used correctly, is an effective, often life-long treatment [11]. The CPAP mask covers the face or mouth and/or nose, and adequate adherence is often defined as 4–5 h/night at least five nights/week [11, 13].

Untreated OSA can affect the quality of life as well as increase the risk for conditions such as systemic hypertension, stroke, and type II diabetes, which makes adherence to treatment essential [14–16]. Difficulties adapting to treatment and side-effects are some of the reasons why approximately 30% of all CPAP users abandon their treatment [17, 18]. Frequently reported side-effects affecting adherence include xerostomia, blocked up nose, mask leaks, and mask pressure [19]. In two studies focusing on oral health related experiences during CPAP treatment as described by CPAP users, several experiences were lifted [20, 21]. Xerostomia, increased mouth-breathing, and a feeling of an deteriorating oral health in general was among the negative experiences [20] as well as excessive saliva, xerostomia, and shifting bites [21]. One of the most reported side-effects of CPAP treatment is xerostomia which has been associated with treatment abandonment within the first year [18, 19, 22–26]. However, to our knowledge, research on the association between oral health and OSA has previously been performed from a biomedical or technical perspective with focus on treatment with oral appliances or periodontal disease, and research on other aspects of oral health and the patient perspective is scarce.

By using the FDI’s framework to explore oral health determinants in a specific population with an increased risk (i.e. due to CPAP-treated OSA) for negative consequences regarding both general and oral health, we can explore how persons with experience of CPAP-treatment view factors that affect their oral health. Creating a common ground for communication could help oral healthcare professionals and CPAP-practitioners to provide person-centred care and could enable shared decision-making within dentistry [27–29].

## Methods

### Aim

To explore what persons with experience of CPAP-treatment view as determinants for their oral health.

### Design

A study with a descriptive and explorative design was adopted and employed, using a qualitative method for data collection and analysis. Data were collected by semi-structured interviews and analysed by directed content analysis [DCA] [30, 31]. Guided by the aim of the study, an inductive approach was used to extract meaning units, then a deductive approach was used during categorisation. The consolidated criteria for reporting qualitative studies [COREQ] checklist was used to ensure comprehensive and structured details were obtained regarding the research process [32].

### Study context

Generally, an improved oral health in the Swedish population during the last 40 years has been reported [33]. Since the introduction of public dental healthcare in the 1930s, the accessibility of dental care has been generally good and since the 1970s, subsidised oral healthcare has been available in different forms [34]. In Sweden during 2017–2019, approximately 80% of the population (≥ 24 years old) and between 83.3–89.5% among 60–80 years old, visited a dental clinic [35]. Regarding OSA and CPAP treatment, in 2020 and 2021 19 582 diagnostic visits were made at sleep centres in Sweden. In the Swedish sleep apnea registry, 34.2% are female and the mean age for females and males are 57 (± 14) years and 54 (± 14) years, respectively. During 2020–21, 50.6% of the patients were recommended CPAP treatment [36].

### The FDI’s description of driving determinants

**Fig. 1:**
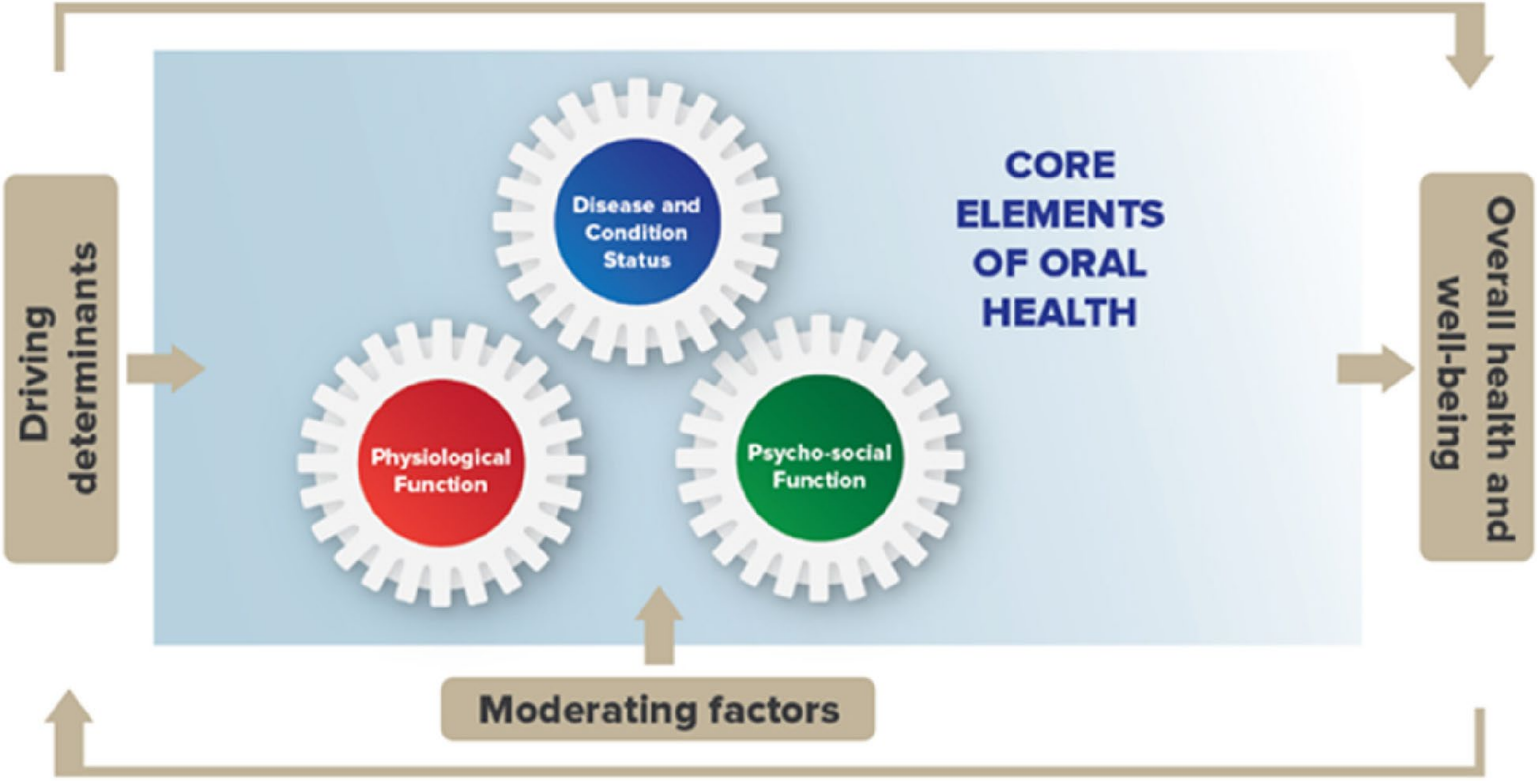
The FDI’s theoretical framework of oral health [1], with permission from the FDI

The FDI’s entire framework illustrates the components of oral health and how complex interactions among driving determinants, moderating factors, and overall health and well-being, together form our oral health, Fig. 1 [2]. The component driving determinants in the FDI’s framework is described as including elements that affect, influence or determine oral health, and is divided into five domains: i) biological and genetic factors, ii) social environment, iii) physical environment, iv) health behaviour, and v) access to care [2].

### Study population

Informants were purposively selected to gain a variety in sex, age, and oral health, based on a sample with hypertensive persons with OSA participating in a longitudinal research project on OSA initiated in 2007. The initial project aimed to study the prevalence of undiagnosed OSA among hypertensive persons in a primary care setting [37]. The current project includes the same hypertensive population, aiming to investigate their oral health. First, the informants were invited to participate in a full dental examination including radiographic examination and to respond to a questionnaire directed towards oral health and sleep (*N* = 121). The examinations were conducted by an experienced dentist and dental hygienist (H.A.), during November 2018 to June 2019. After participating in the initial dental examinations in the project, all hypertensive persons diagnosed with OSA in 2007–2009 with ongoing or previous experience of long-term CPAP-treatment (> 1 year) were asked to participate in the current study (*n* = 42). The informants were interviewed once by using an interview guide designed to capture two different perspectives on oral health in relation to CPAP-treatment and OSA; the present perspective, focusing on views on oral health determinants, and another perspective focusing on critical incidents related to oral health [20]. The final sample consisted of 18 persons where most had > 10 years of experience of CPAP-treatment.

### Data collection procedure

Before the data collection started, the informants were given written and verbal information about the study (e.g. aim, example of questions, and duration). The semi-structured interview guide was developed by the research group, which consisted of professionals with expertise in oral health, sleep medicine, and methodology. The questions were used to direct the persons towards the focus of the interview (i.e. sleep, CPAP-treatment, and oral health), then necessary probing and follow-up questions to obtain comprehensive descriptions of their views were used. Two pilot interviews were conducted with persons in their initial phase of CPAP-treatment (< 1 year) before the data collection started, and the guide was adjusted according to their feedback (minor corrections, e.g. regarding level of detail of follow-up questions). The final version consisted of seven questions (example in Table 1). Data collection was conducted by telephone due to the Covid-19 restrictions during November 2020 and January 2021 by the first author H.A.. The individual interviews lasted approximately one hour (maximum duration two hours) and were recorded.

**Table 1:** Example of questions and follow-up or probing questions from the interview guide

	**Question**	**Follow-up questions (examples)**
Swedish	Berätta om hur du ser på faktorer som påverkar din mun och dina tänder	Du pratade om X, kan du beskriva det mer?
English translation	Tell me about your view on factors that affect your mouth and teeth	Previously you talked about X, could you elaborate on…

### Data analysis

The recordings were transcribed verbatim by the first author H.A. and a research assistant and formed the unit of analysis. To get a sense of the whole, the material (260 pages, Times New Roman 11pt, space 1.15) was then read carefully twice. Data were analysed by DCA [30] and guided by the description of the DCA process by Assarroudis et al. [31]. First, inductive identification of meaning units with a focus on the manifest content of the meaning units was performed by H.A., and all meaning units with relevance to the aim were highlighted in the transcripts [30]. After the initial highlighting of meaning units, this step was repeated to ensure that all important aspects of interest related to the aim were identified. The meaning units were then extracted and carefully condensed to retain the essence of the meaning unit (Table 2). Throughout, discussions within the research group were performed until consensus regarding the identification, extraction, and condensed meaning units, was achieved.

**Table 2:** Illustration of the directed content analysis coding process from meaning unit to category

Meaning unit	Condensed meaning unit	Code	Subcategory	Category
“Yes, yes. I don’t believe in keep on carrying about the nail-scissors or nail file, toothbrush, deodorant, and things like that. It’s better to have more than one, otherwise you will forget them one beautiful day. So that’s…When I take a shower and use the sauna at the gym, of course I brush my teeth. It kind of belongs together.”	I don’t believe in carrying about my hygiene products. It’s better to have several otherwise you will forget them one day. I always brush my teeth when I shower and use the sauna. They belong together	Make it possible wherever you are	One’s motivation to maintain oral hygiene	Health behaviour

**Fig. 2:**
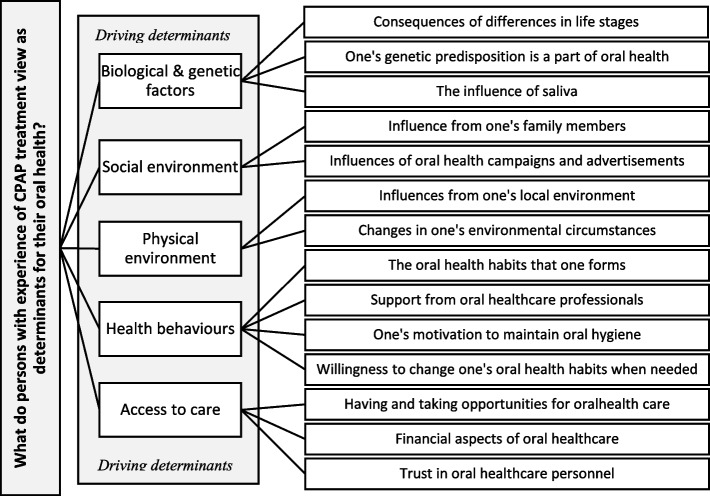
Description of formative categorisation matrix (marked in grey) and final categorisation based on the FDI’s theoretical framework of oral health

The second part of the analysis was performed with a deductive approach. A categorisation matrix was developed based on the FDI’s description of driving determinants. Thus, driving determinants constitute the main area in the categorisation matrix, divided into five pre-determined categories derived directly from the FDI’s framework described above [2]. In the categorisation matrix, content descriptions for each category were developed to facilitate an analysis of the data in this study. By using the categorisation matrix with the case-relevant content descriptions, the condensed meaning units were sorted into the pre-determined categories (Fig. 2). The manifest condensed meaning units were then coded and critically re-evaluated according to the description of the main area and categories in the coding matrix to ensure they were appropriately sorted. In the third part of the analysis, the codes were iteratively compared and clustered into subcategories according to their content (Fig. 2). After repeated critical evaluation and reflection within the research group regarding the coding, consensus was achieved, and the categorisation was considered finalised. In Table 2, a description of the analytical process can be found and verbatim quotations from the informants illustrate the findings in the main text. If needed, they have been shortened (…) or clarified by using […] around the addition.

## Findings

Characteristics of the study population can be found in Table 3. In each of the five domains derived from the FDI’s theoretical framework, which in this study represented the categories, we found subcategories that reflect the views on oral health determinants as experienced by persons with long-term experience of CPAP-treatment. An illustration of the formative and final categorisation is presented in Fig. 2.

**Table 3:** Characteristics of study population

General	(*N* = 18)
Age, md (min–max)	73.5 (51–78)
Sex, n	
Female	5
Male	13
Current occupation, n	
Retired	16
Employed	2
Education within dentistry or general health care, n	
Yes	3
No	15
Medication use, total (> 5 medications), n	
Yes	7
No	11
**AHI before treatment and CPAP adherence**	
AHI-score before CPAP treatment, md (min–max)	23.9 (8.4–74.6)
Self-reported active CPAP user, n	
Yes	17
No	1
Adherence obtained from the CPAP device (> 4 h/night), n	
Yes	15
No	2
CPAP mask type, n	
Nose-mask	14
Full-face	3
Humidification use (self-reported), n	
Regular user	11
Non-regular user	4
Non-user	2
**Oral health**	
Time since last visit to an oral health care clinic, n^a^	
< 1 year- 1 year	15
> 1 year	3
Planned visits/years to an oral health care clinic, n^a^	
< 1	1
1	10
2 or more	7
Type of dental clinic, n	
Private	8
Public	10
Tooth-brushing frequency, n	
≥ 2 times/day	16
≤ 1 time/day	2
Interdental cleaning, n, missing *n* = 1	
Yes, every day	8
Yes, more than 3 times/week	7
Yes, but only when needed	2
Number of main meals/days, md (min–max)	2 (1–3)
Number of snacks/days, md (min–max)	2 (0–3)
Number of sugary containing beverages/foods/days, md (min–max)	2 (0–3)
Usually feels dry in the mouth, n	
Yes	1
No	17
Aesthetic satisfaction, n	
Yes, very satisfied	2
Yes, mainly satisfied	14
No, not mainly satisfied	2
Number of teeth, md (min–max)	25 (15–28)
Decayed surfaces, md (min–max)	0.5 (0–15)
Number of restorations, md (min–max)	13 (6–22)
Number of dental implants, md (min–max)	0 (0–4)
Number of dental crowns, md (min–max))	1 (0–7)
Visual plaque index ^b^, md (min–max)	27.9 (4.0–93.5)
Number of periodontal pockets > 4 mm, md (min–max)	8 (0–42)
Number of periodontal pockets > 6 mm, md (min–max)	1.5 (0–19)
Total score OHIP-14, md (min–max), missing *n* = 1	81 (65–84)

*Key*: *md* median, *AHI* Apnoea Hypopnoea Index (based on polygraph data), *CPAP* continuous positive airway pressure (adherence based on reports from CPAP-device), *OHIP-14* the Swedish version of the abbreviated Oral Health Impact Profile (summarised, response 1–6; higher score indicates less oral health-related impact, maximum value 84)

^a^Due to Covid-19 restrictions some have postponed their visits. Planned visits/year refers to their normal visitation frequency

^b^Visible plaque index according to Silness & Löe

### Oral health determinants during a lifetime

The informants identified several oral health determinants related to different stages in life, described how they can affect each other, and related their experiences to a life course perspective of oral health. Becoming older and being treated with CPAP was described as having an additional effect on their oral health in addition to the determinants they acknowledged from their everyday life. The CPAP-treatment was described to have contributed to negative experiences but was also described as a reason to make positive changes that were beneficial to their oral and general health.

#### Biological and genetic factors

Biological and genetic factors were described as oral health determinants both before and during CPAP-treatment by the informants. They described both positive and negative effects on their oral health, based on determinants from the prenatal stages, adolescence, and finally adulthood.

##### Consequences of differences in life stages

The informants described how the changing circumstances they experienced during life and their consequences affected their oral health in different life stages. From the prenatal stages to the effects of ageing, both consequences that appeared instantly and those that became evident later in life were described. By relating to tooth position (e.g. crowding, or additional teeth) and tooth development, the early life stage was described as an oral health determinant that could affect them throughout life. Ageing per se was also viewed as a determinant, which was exemplified with experiences of increased crowding, tooth wear, and xerostomia in older age. Some considered both their CPAP-treatment and becoming older as part of the cause for their xerostomia.

(…) I don’t think it is just the CPAP [that increases the xerostomia], I try to think that it is, (…) both the CPAP and becoming older (…). (Female,76 years)

##### One's genetic predisposition is a part of oral health

By comparing their own oral health to that of their immediate family and persons in their surroundings, the genetic predisposition was experienced as a determinant. In their experience, some risk factors were more easily modifiable than others, and protective factors such as oral hygiene and dietary habits were factors that could balance a disadvantageous genetic heritage. Based on different genetic predispositions, they assumed that some had a better heritage or were luckier than others.

(…) there are those around me (…), who are extremely careful with their teeth, and brush and keep going on, (…) who have much worse teeth than I've ever had. So, it's not just how you take care [of your teeth] but a little bit of luck and what you have inherited as well. (Female, 64 years)

##### The influence of saliva

The informants considered saliva to affect their teeth and mouth, even if the views on cause and effect of for example xerostomia varied. Experiences of xerostomia during CPAP-treatment could make them contemplate about how saliva could affect their oral health. They also expressed ambivalent thoughts on the cause of their xerostomia; was it due to mouth-breathing during CPAP-treatment or part of becoming older? Their views on the influence of saliva as a determinant were also expressed by making assumptions or perceiving themselves to have a lack of knowledge.

(…) That you get more oral dryness [when using the CPAP], and it is not the same…maybe it releases a bit more…as everything dissolves. (…) and then I wonder if they [the restorations] release more if they are drier or if they calm down and release more if they are wet? (…) (Female, 76 years)

#### Social environment

Views on the social environment from throughout life were described, even if most were derived during childhood. The informants also described close relationships such as the child-parent relationship and influences from the surrounding community in different stages in life as oral health determinants.

##### Influence from one's family members

Experiences from the informant’s own upbringing and how it had affected their oral health in adulthood included descriptions of how beneficial or inappropriate oral health care habits (e.g. oral health hygiene or care visits) were formed during their childhood. Habits obtained in childhood were often viewed to be of major importance for their oral health in adulthood. During adolescence, the social environment also included peers, but their families were still influential. They also described how the social context in their childhood neighbourhood affected their parent’s views on oral health through values in the community, and they said that this contextual effect had changed over time. Becoming a parent or grandparent also made them acknowledge possible differences among families, and how the social environment has contributed to differences among family members. In adulthood, the family constitution had changed but was still viewed as influential, which was exemplified by buying candy from grandchildren or letting their partner make decisions regarding oral hygiene products.

(…) you don’t have to go further than to your own children or grandchildren and compare their oral health to mine. It’s a big difference. (…) But it [oral hygiene habits] can vary between families, I don’t know, I only know how it is with us. (…) (Male, 77 years)

##### Influences from oral health campaigns and advertisements

The societal influence from oral health campaigns and advertisements, for example on social media or television, was viewed by the informants to be influential on their habits and choices. They viewed public oral hygiene or dietary campaigns (e.g. Saturday candy and tooth-brushing twice a day) as influential and described how advertisements could affect their choice of oral hygiene products, especially if the product was promoted by an oral health professional. They acknowledged the wider societal impact on habits such as advertisements of adverse health products in the society, and how making healthy choices nowadays could be difficult.

(…) It’s not that easy today because we are overwhelmed by commercials for a lot of stuff that is not healthy. Unfortunately*.* (Male, 52 years)

#### Physical environment

Oral health determinants related to the physical environment were viewed to have been influential in different ways during their life. Both the actual location and the characteristics of the location were described as determinants, and even a small or inevitable change could be experienced as having a significant impact on their oral health.

##### Influences from one’s local environment during childhood

The informants identified different ways the local environment in their childhood could be viewed as an oral health determinant. Having natural high levels of fluoride in drinking water during childhood was viewed as positive, but also recognised as an increased risk factor for dental fluorosis. Moving away from that location was also considered a risk for development of dental caries. They also acknowledged the influence of growing up in a rural instead of an urban area and said that the differences regarding the local community’s efforts to provide access to oral healthcare clinics and to offer fluoride rinses in school, depended on where they lived.

(…) where I grew up, where I live now as well, there has always been a lot of fluoride (…). It could have been good; it could strengthen the teeth. But then it has been a couple of years in between (…). That it [dental caries] has developed…I have gotten dental caries (…) (Female, 69 years)

##### Changes in one’s environmental circumstances

Changes were described to occur both when various conditions changed in their immediate environment, and when they changed location. CPAP-related xerostomia could for example either increase or decrease in impact due to changes in the local physical environment, and even small changes could have a major impact on their experience. Changes in air humidity, due to seasonal changes or when changing location, were experienced as affecting their xerostomia. The descriptions included experiences of increased xerostomia when using the CPAP during autumn/winter due to decreased air humidity or to frequently having a blocked-up nose. This made it difficult to use the CPAP and thus the xerostomia increased. Circumstances in the surrounding environment were also experienced as varying between different locations, where locations with higher levels of air humidity could decrease the xerostomia. Changing location was also experienced as influencing their oral hygiene habits negatively and made it more difficult to find an oral healthcare clinic if needed.

Yes, but more or less [xerostomia]. But it can be worse some days, some mornings it can be worse (…) for example, if you don’t have the window open, one night, it can be the fresh air, that’s not bad either, it comes in [the bedroom] in one way or another. (…) (Male, 69 years)

#### Health behaviour

The subcategory Health behaviour in this context regarded specifically oral health behaviour and was described in several ways, from creation of oral health habits to maintaining them over time. This was related to the informant’s willingness to make changes when needed and the support they experienced from oral health care personnel. They also described both facilitators and barriers regarding their oral health habits, and how they maintained or improved their oral hygiene during CPAP-treatment.

##### The oral health habits that one forms

The informants described that their oral health habits (e.g. oral hygiene or dietary habits), were formed based on their understanding or knowledge of oral health, and some relied on their formal education within healthcare or oral healthcare. The views on how they gained and viewed their knowledge varied among the informants, but making healthy choices was mostly viewed as important. When they described how their everyday habits were formed, they also weighed previous experiences of negative outcomes against their experienced beneficial habits, such as being a non-smoker, using fluoride toothpaste or an electric toothbrush, and their dietary habits. Relying on hearsay from others in their surroundings sometimes led to increased uncertainty when their views based on hearsay from others contradicted their own experiences.

(…) But I think I heard it from someone that you either have one or the other [dental caries or calculus]. However, sometimes I have noticed that you have both, you [I] had both cavities and calculus (…) (Female, 67 years)

##### Support from oral healthcare professionals

The received information or recommendations from oral health care personnel throughout their life was described as an oral health determinant. It was not always the content of the information, but the experience of getting help or how it was given and received that had influenced their oral health. Receiving support made them view themselves and the oral health care personnel as a team working together, or it could make them feel safe. Receiving feedback on their efforts was viewed as beneficial for their oral health. Support was also described as a feeling that the personnel would not give up on them or that they were thorough, but they also described their own role in the encounter. While some experienced that they had not been pushed enough, others experienced that they had done what they were told or their best, but it did not work. Some described how lack of support or negative experiences affected them as well and described experiences of dentists scolding them or the hygienist whining.

(…) if you [I] had received it [the information] during my 20s you know, it would probably have affected [my oral health] in a different way… (…) (pause). Yes, it affected it [oral health], that I am convinced of, but then if you are receptive to it [the information] it’s like that. Maybe I wasn’t. It’s possible that someone tried, one [I] was not receptive to it [the information] (Male, 77 years)

##### One's motivation to maintain oral hygiene

The informants also described facilitators and barriers in daily life and what motivated them to maintain their habits. The motivation for maintaining adequate oral hygiene habits was often the perceived importance of having good oral health, or the positive sensation within the mouth. This motivation provided oral hygiene habits that felt habitual or a natural part of their daily routine. Lack of motivation was not always the reason for not maintaining adequate habits as other factors acted as barriers. Experiences of feeling nauseous or being uncomfortable when using oral aids, were viewed as ongoing barriers which were hard to overcome. Other barriers could be managed, and the solution was often to make their oral hygiene habits a part of their everyday life, and they incorporated them in other daily activities, for example by performing tooth-brushing or interdental cleaning in other locations than the bathroom. But there were also those who were aware that they could do better but lacked the motivation to maintain or improve their oral hygiene habits, and who described themselves as careless, forgetful, or too comfortable.

(…) Brushing your teeth is, as you say, in the bone marrow, I have always done it (…) (Male, 76 years)

##### Willingness to change one's oral health habits when needed

The informants were often willing to make changes to improve their, oral health habits when they experienced a negative change in their oral health. CPAP-treatment was described as having increased their awareness of their oral health, and the increased xerostomia made them actively change their oral hygiene or dietary habits to decrease the risk of oral disease or loss of teeth. Even if they experienced a need to make changes, some actively chose not to, while others were more passive by not thinking about their oral health. They also described their willingness to take responsibility for their oral health, which was described by claiming that they did their best or by justifying their choice to not make any changes. Sometimes they were willing to make changes, but due to their general health it was impossible. Being aware of potential negative side-effects of for example CPAP-treatment or noticing an improvement when making a change was described as increasing their willingness to maintain the changes they made.

Yes, I have, it is probable that I have understood that there are some risks that when you… with the CPAP the oral hygiene could deteriorate. (…) (Male, 75 years)

#### Access to care

The informant’s views on having access to oral healthcare included other aspects than availability by having a clinic nearby. They also described how for example control, trust in oral health care personnel, and financial or organisational aspects were viewed as elements of access to care.

##### Having and taking opportunities for oral healthcare

Access to care was considered by the informants to be demonstrated by being offered and accepting the offer to visit an oral healthcare clinic, regularly or when needed. Their childhood experiences varied from lack of regular attendance due to lack of availability, to experiences of being a regular attender from an early age which could be related to their parents’ education or profession. Being offered and accepting the offer of regular visits was experienced as facilitating preventive or promotive oral health care and acute visits. Due to lack of availability (e.g. moving around or no clinics in the area), some described previous difficulties facilitating regular visits as adults and only made acute visits. Confirming that one was a regular visitor or choosing to postpone visits were expressed as ways to have control of their oral health. Relying on the clinics’ regular re-call system to send a reminder within pre-decided time intervals, and thereby handing over control of their oral health, was described. However, this could lead to negative feelings such as disappointment or loss of control if their expectations were not met. Negative experiences in the dental encounter could lead to rejecting an offered appointment or changing oral health care provider to take back control of their oral health. Becoming a regular visitor after years of non-attending was experienced as improving oral health as not being a regular visitor was associated with deteriorated oral health. However, some challenged this view, as being a regular attender per se does not provide any information regarding the quality of care.

Otherwise, it is better to go to the dentist a bit more often, then you have more control, you know. (…) It is important. (Male, 76 years)

##### Financial aspects of oral healthcare

Postponing visits was experienced by the informants to lead to more expensive treatments, and regular visits were therefore viewed as more economical. However, no-one said that they had to postpone oral health care due to financial reasons but showed an understanding for those who had to. The cost for oral health care was viewed as inevitable; postponing visits only meant that you paid for treatments instead of preventive care. In relation to this, some of them also questioned the organisational and financial separation of oral and general healthcare. Financial aspects also included experiences of greedy dentists who cared more about making money, as well as organisational aspects regarding for example lack of dental hygienists or having to see a new dentist at every visit.

(…) But I can also understand why some people don’t go, it’s too expensive. That’s why I think it should be included in the regular health insurance system because it is equally as important as the rest of the body (…) (Female, 64 years)

##### Trust in oral healthcare personnel

Having a trusting relationship with oral health personnel was viewed by the informants as important by many of the informants in this study. Experiences from childhood often included descriptions of terrifying visits, which could affect their trust in oral health care personnel for a while in adulthood.

A treatment like that as it was back then. It was forced treatment, because they tied one [me] to the dental chair to make me sit still. And then it was….and the drills (…) they kept going until there was smoke, now it is water cooling so now it doesn’t feel like that. The old drills were driven by big straps in the ceiling, it was a terrible appliance. Just to see the equipment made you, [I felt I] almost died. (Male, 76 years)

However, fearsome experiences as a child or adult, such as harsh dental professionals, and painful or uncomfortable treatments, did not impact their will to visit a clinic today as they had regained trust in their oral health care personnel. Most of them did not view themselves as afraid or anxious, and described themselves as regular visitors, which some related to their ability to adapt and thereby be able to overcome previous experiences. For some, the lack of trust in oral health care personnel made them consider or did change clinic. Meeting the same personnel was described as important for some of them, as it provided continuity and accountability. Some of them said that their trust was restored in adulthood as the oral health care personnel were experienced as thorough, better educated within their area of expertise, and more caring nowadays. Trust was also fostered by having the possibility to participate in the planning of their care, which was also related to having a trusting relationship.

## Discussion

To the best of our knowledge, this study is the first to explore oral health determinants from the perspective of persons with long-term experience of CPAP-treated OSA by using the FDI’s theoretical framework of oral health. The FDI’s definition and theoretical framework [2] was useful to describe and categorise the variety of views of oral health determinants that were expressed by the persons in the study. The qualitative data also added descriptions of some complex interactions between determinants and the informants’ experiences of adapting to changes in their life situation. Previously, studies have focused on and shown that OSA and oral diseases are associated with biomedical factors (e.g. through systemic inflammation) such as cardiovascular diseases and metabolic disorders e.g. obesity and diabetes mellitus [3, 5, 11], and that xerostomia is a common side-effect of CPAP-treatment [18, 19]. Due to the increased risk for adverse oral health outcomes for this patient group, the knowledge provided in this study can hopefully increase the understanding of oral health during CPAP-treatment for oral healthcare personnel and CPAP-practitioners.

Early life experiences, genetic predisposition, and saliva were viewed as influential oral health determinants which could have immediate consequences or affect the informant’s oral health throughout life. Their views correspond to the consensus report on the interactions among lifestyle, behaviour, and systemic and oral diseases [38], where genetic predisposition and acquired risk factors for dental caries and periodontal diseases were described. The informants considered their saliva to affect their oral health, but the underlying reasons were often considered to be more unclear. They associated their experience of xerostomia with their CPAP-treatment but also described more complex interactions among their treatment, becoming older, and breathing through their mouth. This ambivalence regarding the cause of xerostomia is understandable, as there can be several causes, such as increasing age, medication use, and systemic diseases [39, 40]. However, xerostomia has been associated with OSA [22, 41] and is a commonly reported side-effect of CPAP-treatment that can affect adherence [19, 42]. Therefore, it is important that both CPAP-practitioners and oral healthcare personnel can identity signs and possible cause of xerostomia to provide adequate treatment to prevent adverse oral health outcomes and to reduce or eliminate the person’s difficulties.

The community and the immediate family were described as influential throughout life on the informants’ oral health, from childhood until today. The parental influence and communicative behaviour have previously been shown to affect children’s understanding of oral health and their oral health behaviour [43], and in a study on independent Canadian elderly, oral healthcare utilisation habits were shown to be formed during childhood and to continue though adulthood as normative behaviour [44]. In this population, understanding of the impact of their upbringing on their oral health habits was seemingly of specific importance. Establishing appropriate oral health behaviour at an early age, considering the child and the living social environment (e.g. family, community) [45], is of importance for oral health outcomes in later life. The findings also showed the value of community-driven programmes promoting oral health in adulthood, such as oral healthcare campaigns on tooth-brushing and diet.

Besides the views on influential factors in the informants’ childhood, changes in the local environment in adulthood during CPAP-treatment were viewed to have an impact on their oral health, such as the experience of xerostomia. They connected this with changes in air humidity and increased prevalence of having a blocked-up nose due to seasonal changes. Previously, seasonal changes (i.e. air humidity) have been reported to affect CPAP-adherence in a Japanese population [45] where most of the persons adhered to their treatment; however, possible seasonal changes in adherence were not further explored in this study. In addition to changes in the level of xerostomia, the informants in the current study also described how a change in location could affect their oral hygiene. The findings suggest that circumstantial changes in the environment could affect the level of xerostomia, CPAP-adherence, and oral health habits in this population.

Motivation and willingness to change affected the informant’s behaviour, where support from oral healthcare personnel contributed either as a facilitator or barrier. CPAP-treatment was viewed to have increased their awareness of their oral health and therefore changed their oral hygiene habits to prevent adverse oral health outcomes. As CPAP-treatment is often life-long, it is important for CPAP-users to maintain positive changes in oral health behaviour for a long time. Previously, it has been reported that several factors (e.g. emotions, motivation, and functional and cognitive abilities) can affect a person’s ability to maintain adequate oral hygiene with increasing age [46]. The informants described different views on their willingness and ability to change. In view of these individual differences in motivation for change, person-centred care seems appropriate [28]. This means that oral healthcare personnel may need to maintain or create more trusting oral healthcare encounters and develop shared decision-making further within their clinical context, to accommodate personalised recommendations [29]. Furthermore, the informants’ views on access to oral healthcare were described as a wider concept than just availability. The informants included aspects such as control, finances, organisation, and trust. Creating a trusting environment is important as trusting relationships have previously been described to increase the utility of services, increase adherence to preventive care, and enable oral health care personnel to carry out high quality care [44, 47]. However, there seems to be a lack of knowledge or experience of patients with OSA and the association with oral health among oral healthcare personnel [48].

Based on the findings in this study some recommendations for clinical practice can be highlighted. The knowledge provided in this study could be useful in both identifying oral health needs as well as tailoring oral healthcare to this specific population. Person-reported information regarding oral health related barriers and facilitators for CPAP-treatment adherence is therefore significant. For oral health care personnel, it is important to create trustful relationships to promote beneficial oral health habits including regular visits over time, further implement shared-decision-making, and increase interprofessional collaboration with CPAP-practitioners. For CPAP-practitioners, identifying oral health-related side-effects of CPAP-treatment and providing adequate recommendations can be a challenge as studies on the patient perspective on oral health during CPAP-treatment are scarce. Therefore, it is suggested that future studies focus on the patient perspective on real-life oral health-related experiences before and during CPAP-treatment. However, until then, CPAP-practitioners could ask their patients regarding known side-effects such as xerostomia as a first step to include oral health within CPAP care.

Finally, some methodological considerations should be noted. All persons eligible to participate in the interviews (i.e. participated in the initial dental examinations and with experience of CPAP treatment) were invited to participate. Of the potential 42 informants, 18 persons chose to participate which was regarded as sufficient based on the predetermined aim of 15–20 informants. The informants had previously participated in research projects and were familiar with the research context. In addition, the informants had also met the interviewer (first author, who is a dental hygienist) in person before and/or been in contact with her per telephone which was clarified in the information letter. In our study, this enabled in-depth descriptions and had a positive effect on the informants’ willingness to share their experiences. By the initial inductive approach, all views from the informants were considered for inclusion. The following deductive coding process allowed us to describe the determinants in a comprehensive way while still maintaining focus on the views described by the informants. Although face-to-face interviews, as originally planned, would have been optimal, telephone interviews are common and accepted as a method for data collection [49]. But even if this way of collecting data increase the risk of missing non-verbal forms of communication, the focus in this study was on the manifest content making telephone interviews a suitable method for data collection. The following measures were taken to increase the trustworthiness of the findings [50, 51]. The procedure of purposeful selection of 18 informants resulted in a variety of views of persons with different real-life experiences, which ensured the sample’s heterogeneity. However, more male than female informants participated in the study which probably is due to the differences in prevalence of OSA [11]. This should be considered when assessing the transferability to other contexts. Furthermore, several measures were taken to increase the credibility of the study. First the interview guide was discussed and revised by the research group, consisting of persons with experience and expertise within oral health and sleep medicine, but also methodology, several times. Then pilot interviews and pre-tests were performed to assess the relevance for the area of interest and to ensure an adequate item sequence during the interviews. We also aimed to provide detailed information (e.g. study context, analytic process) to increase the credibility of the findings. Throughout, repeated discussions within the research group, who had access to all material during the analytic process, were performed until consensus was achieved.

## Conclusion

The qualitative data reflected the informants’ views on oral health determinants, which were related to all five dimensions of the FDI´s driving determinants for oral health. Long-term CPAP-treatment can increase the awareness of one’s oral health and improve oral hygiene habits, but can also induce xerostomia, and can therefore be considered an oral health determinant that interacts with other determinants related to the physical environment and health behaviour. The study points to a variety of individual oral health-related experiences worth considering when designing interventions to reduce xerostomia and prevent adverse oral health outcomes for persons with long-term CPAP-treatment.

## Data Availability

The data material generated and analysed during the current study are not publicly available due to privacy or ethical restrictions. Requests regarding the data that support the findings of this study can be sent to the corresponding author (H.A.).

## Abbreviations

COREQ: Consolidated criteria for reporting qualitative studies
CPAP: Continuous positive airway pressure
DCA: Directed content analysis
FDI: World dental federation
OSA: Obstructive sleep apnoea

## References

[1] Glick M, Williams DM. Vision 2020. A new definition for oral health: World Dental Federation [FDI]. 2017. Available from: http://www.fdiworlddental.org/sites/default/files/media/images/oral_health_definition-exec_summary-en.pdf.

[2] Glick M, Williams DM, Kleinman DV, Vujicic M, Watt RG, Weyant RJ (2017). Reprint of: a new definition for oral health supported by FDI opens the door to a universal definition of oral health. J Dent.

[3] Jepsen S, Caton JG, Albandar JM, Bissada NF, Bouchard P, Cortellini P, et al. Periodontal manifestations of systemic diseases and developmental and acquired conditions: consensus report of workgroup 3 of the 2017 world workshop on the classification of periodontal and peri-implant diseases and conditions. J Clin Periodontol. 2018;45(S20):S219–29. 10.1111/jcpe.12951 29926500

[4] Sanz M, Marco Del Castillo A, Jepsen S, Gonzalez-Juanatey JR, D’Aiuto F, Bouchard P (2020). Periodontitis and cardiovascular diseases: consensus report. J Clin Periodontol.

[5] Albandar JM, Susin C, Hughes FJ (2018). Manifestations of systemic diseases and conditions that affect the periodontal attachment apparatus: case definitions and diagnostic considerations. J Periodontol.

[6] Peres MA, Macpherson LMD, Weyant RJ, Daly B, Venturelli R, Mathur MR (2019). Oral diseases: a global public health challenge. Lancet (London, England).

[7] Watt RG, Sheiham A (2012). Integrating the common risk factor approach into a social determinants framework. Commun Dent Oral Epidemiol.

[8] Acar M, Türkcan I, Özdaş T, Bal C, Cingi C (2015). Obstructive sleep apnoea syndrome does not negatively affect oral and dental health. J Laryngol Otol.

[9] Tremblay C, Beaudry P, Bissonnette C, Gauthier CA, Girard S, Milot MP (2017). Periodontitis and obstructive sleep apnea: a literature review. J Dent Sleep Med.

[10] Al-Jewair T, Apessos I, Stellrecht E, Koch R, Almaghrabi B (2020). An update on the association between periodontitis and obstructive sleep apnea. Curr Oral Health Rep.

[11] Gottlieb DJ, Punjabi NM (2020). Diagnosis and management of obstructive sleep apnea: a review. J Am Med Assoc.

[12] Benjafield AV, Ayas NT, Eastwood PR, Heinzer R, Ip MSM, Morrell MJ, et al. Estimation of the global prevalence and burden of obstructive sleep apnoea: a literature-based analysis. Lancet Respir Med. 2019;7(8):687–98. 10.1016/S2213-2600(19)30198-5 PMC7007763 31300334

[13] Chai-Coetzer CL, Pathinathan A, Smith BJ. Continuous positive airway pressure delivery interfaces for obstructive sleep apnoea. Cochrane Database Syst Rev. 2006(4):1–24. 10.1002/14651858.CD005308.pub2 PMC8883876 17054251

[14] Franklin KA, Lindberg E (2015). Obstructive sleep apnea is a common disorder in the population—a review on the epidemiology of sleep apnea. J Thorac Dis.

[15] Garbarino S, Lanteri P, Durando P, Magnavita N, Sannita WG (2016). Co-morbidity, mortality, quality of life and the healthcare/welfare/social costs of disordered sleep: a rapid review. Int J Environ Res Public Health.

[16] Strausz S, Havulinna AS, Tuomi T, Bachour A, Groop L, Mäkitie A (2018). Obstructive sleep apnoea and the risk for coronary heart disease and type 2 diabetes: a longitudinal population-based study in Finland. BMJ Open.

[17] Rotenberg BW, Murariu D, Pang KP (2016). Trends in CPAP adherence over twenty years of data collection: a flattened curve. J Otolaryngol Head Neck Surg.

[18] Broström A, Nilsen P, Johansson P, Ulander M, Strömberg A, Svanborg E (2010). Putative facilitators and barriers for adherence to CPAP treatment in patients with obstructive sleep apnea syndrome: a qualitative content analysis. Sleep Med.

[19] Ulander M, Johansson MS, Ewaldh AE, Svanborg E, Broström A (2014). Side effects to continuous positive airway pressure treatment for obstructive sleep apnoea: changes over time and association to adherence. Sleep Breath.

[20] Ahonen H, Broström A, Fransson EI, Neher M, Lindmark U. "The terrible dryness woke me up, I had some trouble breathing"-Critical situations related to oral health as described by CPAP-treated persons with obstructive sleep apnea. J Sleep Res. 2022;31(6):e13670. 10.1111/jsr.13670 PMC10909513 35765213

[21] Almeida FR, Henrich N, Marra C, Lynd LD, Lowe AA, Tsuda H (2013). Patient preferences and experiences of CPAP and oral appliances for the treatment of obstructive sleep apnea: a qualitative analysis. Sleep & Breathing.

[22] Makeeva IM, Budina TV, Turkina AY, Poluektov MG, Kondratiev SA, Arakelyan MG, et al. Xerostomia and hyposalivation in patients with obstructive sleep apnea. Clin Otolaryngol. 2021;46(4):782–87. 10.1111/coa.13735 33548090

[23] Oksenberg A, Froom P, Melamed S (2006). Dry mouth upon awakening in obstructive sleep apnea. J Sleep Res.

[24] Broström A, Strömberg A, Ulander M, Fridlund B, Mårtensson J, Svanborg E (2009). Perceived informational needs, side-effects and their consequences on adherence—a comparison between CPAP treated patients with OSAS and healthcare personnel. Patient Educ Couns.

[25] Almeida FR, Henrich N, Marra C, Lynd LD, Lowe AA, Tsuda H (2013). Patient preferences and experiences of CPAP and oral appliances for the treatment of obstructive sleep apnea: a qualitative analysis. Sleep and Breathing.

[26] Tsuda H, Moritsuchi Y, Higuchi Y, Tsuda T (2016). Oral health under use of continuous positive airway pressure and interest in alternative therapy in patients with obstructive sleep apnoea: a questionnaire-based survey. Gerodontology.

[27] Walji MF, Karimbux NY, Spielman AI (2017). Person-centered care: opportunities and challenges for academic dental institutions and programs. J Dent Educ.

[28] Lee H, Chalmers NI, Brow A, Boynes S, Monopoli M, Doherty M (2018). Person-centered care model in dentistry. BMC Oral Health.

[29] Charles C, Gafni A, Whelan T (1997). Shared decision-making in the medical encounter: What does it mean? (or it takes at least two to tango). Soc Sci Med.

[30] Hsieh HF, Shannon SE (2005). Three approaches to qualitative content analysis. Qual Health Res.

[31] Assarroudi A, Heshmati Nabavi F, Armat MR, Ebadi A, Vaismoradi M (2018). Directed qualitative content analysis: the description and elaboration of its underpinning methods and data analysis process. J Res Nurs.

[32] Tong A, Sainsbury P, Craig J (2007). Consolidated criteria for reporting qualitative research (COREQ): a 32-item checklist for interviews and focus groups. Int J Qual Health Care.

[33] Norderyd O, Koch G, Papias A, Anastassaki Köhler A, Nydell Helkimo A, Brahm C-O (2015). Oral health of individuals aged 3–80 years in Jönköping, Sweden during 40 years (1973–2013). Swed Dent J.

[34] Ordell S, Söderfeldt B (2009). Understanding politics? Some lessons from Swedish dentistry. Community Dent Health.

[35] National Board of Health and Welfare. Statistics on dental health 2019. Art.no: 2020-9-6940. Stockholm; 2020.

[36] Grote L, Anderberg C-P, Friberg D, Grundström G, Hinz K, Isaksson G (2023). National knowledge-driven management of obstructive sleep apnea—the Swedish approach. Diagnostics.

[37] Broström A, Sunnergren O, Årestedt K, Johansson P, Ulander M, Riegel B (2012). Factors associated with undiagnosed obstructive sleep apnoea in hypertensive primary care patients. Scand J Prim Health Care.

[38] Chapple ILC, Bouchard P, Cagetti MG, Campus G, Carra MC, Cocco F (2017). Interaction of lifestyle, behaviour or systemic diseases with dental caries and periodontal diseases: consensus report of group 2 of the joint EFP/ ORCA workshop on the boundaries between caries and periodontal diseases. J Clin Periodontol.

[39] Murray TW (2014). Epidemiology of oral health conditions in older people. Gerodontology.

[40] Niklander S, Veas L, Barrera C, Fuentes F, Chiappini G, Marshall M (2017). Risk factors, hyposalivation and impact of xerostomia on oral health-related quality of life. Braz Oral Res.

[41] Broström A, Sunnergren O, Johansson P, Svensson E, Ulander M, Nilsen P (2012). Symtom profile of undiagnosed obstructive sleep aponea in hypertensive outpatients in promary care: a structural equation model analysis. Qual Prim Care.

[42] Rotty MC, Suehs CM, Mallet JP, Martinez C, Borel JC, Rabec C (2021). Mask side-effects in long-term CPAP-patients impact adherence and sleepiness: the InterfaceVent real-life study. Respir Res.

[43] Matsuo F, Sato S, Moriyama M. The effect of parents’ oral health behaviors on children and mutual communication. Pediatr Dent J. 2016;26(3):122–8.

[44] Khabra K, Compton S, Keenan L (2017). Independent older adults perspectives on oral health. Int J Dental Hygiene.

[45] Fujino Y, Oka Y, Wakamura T (2021). Seasonal effects on the continuous positive airway pressure adherence of patients with obstructive sleep apnea. Sleep Med.

[46] Grönbeck Lindén I, Hägglin C, Gahnberg L, Andersson P (2017). Factors affecting older persons’ ability to manage oral hygiene: a qualitative study. JDR Clin Transl Res.

[47] Yamalik N (2005). Dentist-patient relationship and quality care 2. Trust Int Dent J.

[48] Berggren K, Broström A, Firestone A, Wright B, Josefsson E, Lindmark U. Oral health problems linked to obstructive sleep apnea are not always recognized within dental care—as described by dental professionals. Clin Exp Dent Res. 2021;8(1):84–95. 10.1002/cre2.517 PMC8874038 34791818

[49] Novick G (2008). Is there a bias against telephone interviews in qualitative research?. Res Nurs Health.

[50] Graneheim UH, Lundman B (2004). Qualitative content analysis in nursing research: concepts, procedures and measures to achieve trustworthiness. Nurse Educ Today.

[51] Elo S, Kääriäinen M, Kanste O, Pölkki T, Utriainen K, Kyngäs H (2014). Qualitative content analysis: a focus on trustworthiness. SAGE Open.

